# Student Preference on Teaching Mode and the Impact of Remote Teaching on Academic Performance in Undergraduate Orthodontics Course, a Follow‐Up Study

**DOI:** 10.1002/jdd.13995

**Published:** 2025-07-16

**Authors:** Heidi Arponen, Emma Juuri

**Affiliations:** ^1^ Department of Oral and Maxillofacial Diseases University of Helsinki Helsinki Finland; ^2^ Helsinki University Hospital Head and Neck Center Helsinki Finland; ^3^ Department of Plastic Surgery, Cleft Palate and Craniofacial Center University of Helsinki and Helsinki University Hospital Helsinki Finland

**Keywords:** dental education, distance learning, remote teaching, student well‐being, orthodontics

## Abstract

**Objectives:**

The objective of this interventional mixed‐methods study was to evaluate predoctoral dental students’ preferences for different teaching modalities, examine the impact of remote teaching on academic performance, and assess students’ well‐being.

**Methods:**

We implemented a blended teaching approach for the 19 theoretical lectures of an orthodontics course, combining remote delivery for half of the lectures with in‐class sessions for the other half. In total, 51 students participated in the course. To assess learning outcome, the results of a summative course examination comprising 38 multiple‐choice questions were analyzed, and performance on questions related to remotely delivered lecture topics were compared with topics presented through in‐class lectures. The students’ preferences were assessed with a feedback questionnaire and their burnout level with the School Burnout Inventory (SBI‐9). The correlation between teaching method of each topic and the corresponding examination results were investigated to identify for a possible association.

**Results:**

We found that online lectures yielded equally good overall learning outcomes as in‐class lectures. Majority of the students favored the blended teaching approach as they considered that inclusion of remote teaching alleviated their study load and increased well‐being. The students exhibited levels of exhaustion from schoolwork and feelings of inadequacy that were above the SBI‐9 scale average, whereas cynicism toward the value of studies was found to be below the scale average.

**Conclusions:**

A blended teaching approach is effective in theoretical orthodontics and preferred by undergraduate students. This study provides insight for educators to enhance educational outcome and student's well‐being.

## 1 Introduction

Recent educational research has extensively explored various modes of teaching and their effectiveness in higher education [1, 2, 3, 4]. Video and live online teaching and blended teaching approach have been found to stimulate effective learning in dental education and yield good learning results, particularly early in the study progression [5, 6, 7, 8]. However, dental students consider remote learning to be less interactive and prefer learning activities and assessments to be delivered in‐class [6]. The existing literature covers a variety of technologies assisting remote teaching in higher education, such as video conferencing tools, learning management systems, collaboration tools, virtual reality, and screen recording, while limited number of studies address the effects of remote teaching of orthodontics on academic performance of undergraduate students [4, 9, 10, 11].

Postgraduate orthodontic students and teaching staff view blended teaching approach as a stimulating and cost‐effective addition to traditional courses [2]. Postgraduate orthodontic students prefer theoretical courses to be taught remotely, while they believe practical skills are best learned through traditional classroom instruction [3]. Moreover, undergraduate orthodontic students consider web‐based material as a significant supplementary learning resource, but not as a replacement for traditional teaching methods [12]. Video recorded and live lectures have been found to be more effective than audio recorded lectures in promoting higher levels of analytical thinking among undergraduate orthodontic students [13]. Incorporating game‐based learning into both undergraduate and postgraduate education has been associated with improved learning results [14].

The need to promote mental health and well‐being among young university students has been affirmed by several studies, and educational institutions have recognized the need to increase attention to providing support [15]. Burnout, defined by WHO as a syndrome “resulting from chronic workplace stress that has not been successfully managed” [16], has a high prevalence up to 88% among medical students of different countries as demonstrated by a recent review [17]. Several factors contribute to university students’ well‐being and mental load, including learning experience, relationships between students and staff, and study progression [18, 19, 20]. According to Salmela‐Aro, low academic achievement and school engagement of adolescents correlate with high levels of exhaustion, cynicism, depressive symptoms, and feelings of inadequacy [21]. Four profiles of university students have been identified: engaged (positive engagement and low burnout symptom), engaged‐exhausted (students showing exhaustion simultaneously with academic engagement), inefficacious (heightened feeling of inadequacy), and burned‐out (high cynicism and inadequacy, low academic engagement) [22]. Inversely, burnout impairs cognitive functioning, motivation, and emotional well‐being, all of which are essential for academic success [23]. Adjustment and burnout in the study context are significant concerns, as research has shown that it can contribute to worse academic achievement, dropping out of studies or developing depression [23, 24, 25].

This interventional study aimed to evaluate undergraduate dental students’ opinion on preferred teaching mode and the impact of remote teaching on academic performance as assessed by student feedback and examination performance. An additional goal of this study was to assess the presence of burnout symptoms, potentially affecting academic achievement, among the participants.

## 2 Materials and Methods

The research ethics committee of the Faculty of Medicine at the University of Helsinki had approved the study (17/2024). The study hypothesis was that remote teaching is equally preferred by the students as in‐class teaching, and that the teaching mode is not associated with examination success.

This cross‐sectional mixed‐methods study examined participants of an undergraduate orthodontic course conducted during dental students’ third study year within a 5‐month period spanning from August to December in 2024. Inclusion criterion for the study was course participation. In total, 52 students enrolled in the course. One student was excluded from the study due to discontinuation of the course participation. At the beginning of the course, the students received written information outlining the study protocol and were informed of their right to opt out of having their course data included in the study. The current study follows similar protocol to our recent pilot study, [4] conducted during the previous academic year, enabling longitudinal comparison and thereby enhancing the generalizability of the present findings. Three additional courses on other dental disciplines were conducted simultaneously with the orthodontics course under investigation. The course constitutes the majority of orthodontic curriculum provided in the predoctoral dental education at University of Helsinki. The main topics of the course are craniofacial growth, development of the occlusion, etiology and classification of malocclusions, clinical examination and diagnosis, cephalometrics, cellular basis of tooth movement, managing developing dentition, fixed and removable orthodontic appliances, missing and impacted teeth in orthodontics, orthodontic first aid, and screening of malocclusions in public health care (Table S1). Course objectives had been established following the Bloom's taxonomy framework [26]. All the main course topics were categorized in the course learning objectives as essential knowledge requiring higher‐order skills. Learning objectives were available for the students to see throughout the course. The course is mandatory for all students and includes theoretical lectures, workshops, a video lecture, and simulation laboratory teaching facilitated by six experienced university lecturers. Of the 19 lectures included in the course, eight (42%) require compulsory attendance. Student attendance at all teaching sessions was recorded either through a digital attendance system or by manually signed attendance sheets. The students were familiar with Moodle learning platform and had limited previous experience with blended learning.

Before the onset of the course, lectures were systematically allocated by the authors H.A. and E.J. to either remote or in‐class in an alternating pattern, where every other lecture, in chronological order, was designated as a remote lecture and every other as in‐class lecture. Remote lectures were delivered on Zoom platform. An exception of the allocation pattern was made on 2 days, where several lectures were scheduled. The same lecture delivery mode was decided for the whole day, to make studying more convenient for the students. Sample allocation was balanced thereafter so that eventually nine out of the 19 lectures were delivered remotely as a live online lecture and one as a video recording of the lecture. Delivery of both the in‐class and online formats was provided by the same teaching staff. Four of the remote lectures and five of the in‐class lectures were mandatory. The lecture structure consisted of context and learning objectives, content, and closure. The duration of both classroom and live online lectures varied between 45 and 90 min per session. The video recorded lecture duration was 20 min, and it could be re‐watched multiple times. For all lectures, the students were provided with a handout material comprised the main points of the lecture. If a student missed a mandatory lecture, they were required to hand in a written assignment on the subject to verify reaching of the learning objectives of the class.

Additional reading material, videos, and interactive HTML5 educational game contents were provided for 11 of the teaching sessions (61%) in the course's Moodle learning platform. To support self‐guided learning, the activity completion‐function was used in Moodle with the additional material to allow for the students to monitor their progress throughout the course.

The summative course examination consisted of 38 single best answer multiple choice questions with five answer options, that were consistently constructed by the authors H.A. and E.J. in accordance with the principles outlined in The National Board of Medical Examiners item‐writing manual and following the recommendations by Walsh et al. [27, 28]. Each lecture and topic were represented by two questions, which were aligned with the course's learning objectives and teaching content to strengthen validity. Each question had only one correct answer that awarded one point each. The questions contained minimal amount of background information and followed a consistent style. The students’ academic performance was assessed as examination result outcome. The examination questions used in this study were purposefully developed as new unvalidated items to ensure novelty and prevent potential bias associated with prior exposure as the risk of participants preparing in advance by memorizing pre‐existing questions was eliminated. While this choice limited the ability to confirm the psychometric properties of the questions, it was deemed appropriate for the context to ensure authentic evaluation of participants' knowledge and problem‐solving skills. The examination was completed in‐class utilizing Safe Examination Browser program which prohibits the use of online browsing. The examination had a maximum completion time of 3 hours and a one‐attempt limit.

Students’ opinions on and preferences regarding different teaching methods for enhancing learning were collected at the end of the course through an unvalidated anonymous online questionnaire. Responding to the questionnaire was optional following the University of Helsinki policy on course feedback. The questionnaire included 17 multi‐choice questions, of which six were fixed university‐level questions. The fixed questions covered students’ overall perspectives on the course, and they are systematically applied to guide teaching management, decision making, and support the development of teaching practices (Supporting Information file). Preference of teaching mode was inquired with two questions, designed to elicit students’ general views on remote learning. The questions were as follows: “My learning is best supported by classroom teaching/remote teaching/blended teaching,” “My workload is best reduced by classroom teaching/remote teaching/blended teaching/I don't know.” The questionnaire included an option to leave open‐ended feedback.

The University of Helsinki offers to all students a range of mental health related services, including professional support for study‐related challenges and organized peer support. To assess the students’ overall mental workload and burnout symptoms at course completion, the validated School Burnout Inventory (SBI‐9), previously adapted to and successfully used in higher education, was included in the questionnaire [22, 29]. SBI‐9 measures three domains: exhaustion at school, cynicism towards meaning of school, and sense of inadequacy at studies [21]. The answers are given in a six‐point rating Likert scale. Each item is scored between 1 and 6, where the lowest possible sum score of all items is 9, indicating no burnout, and the highest score of 54 is indicative of burnout.

### 2.1 Statistical Analysis

Statistical analyses were performed using SPSS software 23.0 (IBM Corp., NY, USA). Continuous variables (number of attendants, examination scores) and categorical variables (teaching mode, lecture attendance requirement, and SBI‐9 scores) were presented as means, medians, and percentages. The Shapiro–Wilk normality test was used to determine the normal distribution of the variables. The association between examination scores (dependent variable) and lecture attendance, different teaching delivery modes, lecture attendance requirement, and user activity of the additional online material (independent variables) were examined with Spearman's rank correlation as a complete case analysis to calculate the effect size. Mann–Whitney *U*‐test was conducted to compare the distributions of examinations scores between the different lecture groups. A value of *p* < 0.05 was considered statistically significant. Observations with missing data were excluded.

## 3 Results

Our investigation included all 51 dental students, who completed the course and comprised the target population, which ensured that the findings were fully representative of this group and no sampling bias was present. Out of the included study subjects 13 were male and 38 were female. While the small sample size limits statistical power and transferability of the findings, the inclusion of the entire cohort eliminates the need for power calculations and allows for a diverse range of insight and experiences for qualitative analysis.

### 3.1 Lecture Attendance

**FIGURE 1:**
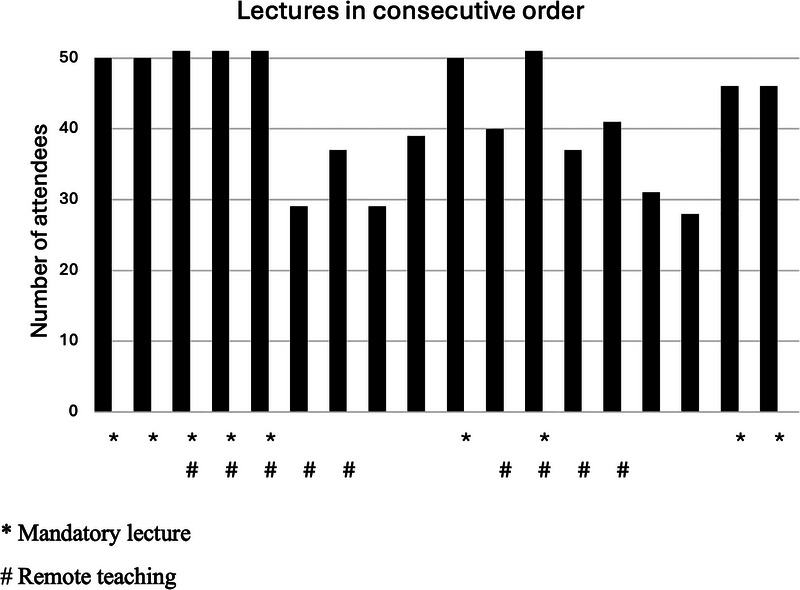
Lecture attendance of 51 students in the 18 lectures of the course (data on one lecture of the course is missing).

The overall average participation rate in the lectures was 82%. Figure 1 presents the attendance rate for the 18 lectures. The lecture participation rate did not correlate with lecture delivery mode (*r*_s_ = 0.129, *p* = 0.609, *n* = 18). Record on the number of attending students was missing from one optional in‐class lecture. Four of the mandatory lectures were attended by all the students (100%) while the average attendance rate across the course was 97% for the mandatory lectures and 68% for the optional lectures. Participation rate was lowest for an optional lecture on the topic of “Retention following orthodontic treatment” (55%). The number of lectures attended by each student ranged from 7 to 17.

### 3.2 Academic Performance

**FIGURE 2:**
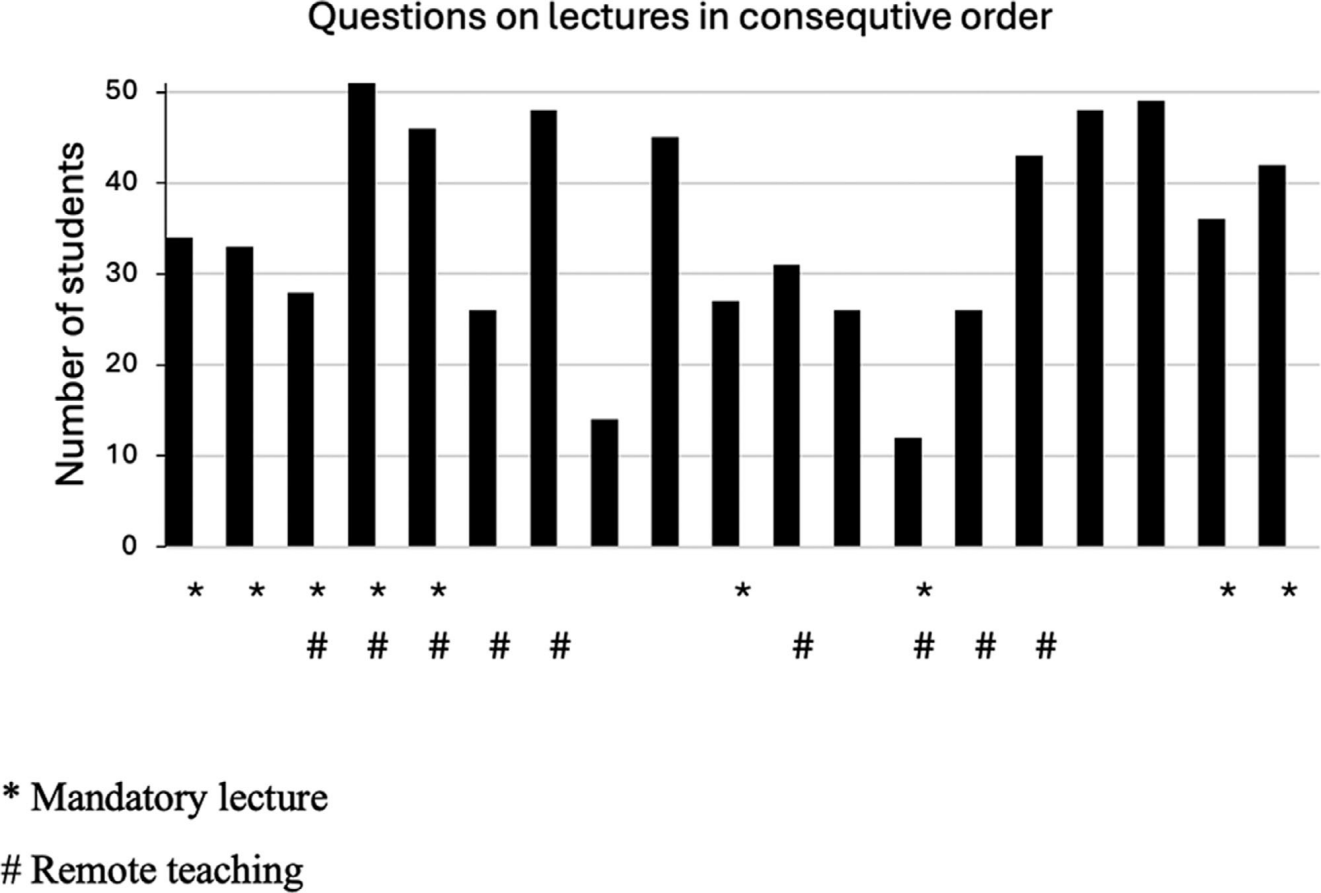
The number of students (out of 51) who correctly answered both examination questions for each topic, categorized by topic.

Overall, considering all the 38 questions of the examination, the examination scores of different topics were not associated with either the lecture delivery mode or the attendance requirement of the lecture (*r*_s_ = 0.044, *p* = 0.795, *n* = 38 and *r*_s_ = 0.061, *p* = 0.718, *n* = 38 respectively) (Figure 2). The number of students with a correct answer to both questions on each of the 19 topics was higher in those topics taught in‐class as compared to the topics taught remotely (*r*_s_ = −0.492, *p* = 0.032, *n* = 19), indicating a meaningful association although the difference did not reach statistical significance (*U* = 42.5, *p* = 0.842). The number of students with a correct examination response was highest in cephalometrics (51 students) and orthodontic retention (49 students), and lowest in treatment of crossbite (14 students) and missing or impacted teeth (12 students).

The average examination score was 31 out of 38. The highest score attained was 37 and the lowest was 22. Examination scores of individual students did not correlate statistically significantly with lecture attendance frequency of the student (*r*_s_ = 0.205, *p* = 0.148, *n* = 51). However, the effect size suggests a small to moderate positive relationship between lecture attendance and high examination scores, indicating that there may be a meaningful relationship.

### 3.3 Descriptive Learning Analytics

On average, 82% of the students used the additional electronic learning material provided in the Moodle learning platform. The additional reading material was visited on average by 32 students (range 13–46 students per material), videos were viewed on average by 36 students (range 5–51 students per video), interactive H5P games were played by an average of 45 students (range 37–51 students per game), and the learning outcomes of the course were viewed by 14 students. The additional material on topic of “Orthodontic first aid” was the least profited (37%) even though the topic was listed as one of the primary learning objectives. The interactive game on identifying orthodontic appliances was the most popular (100% usage rate). The use of the self‐guided study material remained consistent throughout the course (*p* = 0.507) and did not correlate with lecture attendance rate (*r*_s_ = 0.185, *p* = 0.194, *n* = 51). The use of self‐guided study material positively correlated with total examination score, but the association did not reach statistical significance (*r*_s_ = 0.271, *p* = 0.055, *n* = 51).

### 3.4 Subjective Preferences and Wellbeing

In total, 49 students (94%) responded to the feedback questionnaire at the end of the course. Of them, blended teaching approach was preferred by 86%, while 8% would prefer complete remote teaching and 20% would prefer classroom teaching. Furthermore, 55% reported that blended learning reduces studying load, whereas 37% felt that remote teaching, and 6% considered that in‐class teaching reduces study load most effectively. Of the students, 29% agreed or fully agreed that additional online learning material supports their learning process. The statement was partly agreed by 45% and disagreed either partly or completely by 27%. Table 1 outlines the themes of the open‐ended feedback.

**TABLE 1:** Qualitative feedback summary.

Theme	Example comments	Frequency	Summary
**Scheduling issues**	Schedules often not followed; conflicting schedules between platforms; overlap with microbiology course; insufficient time before exams.	10	Many students reported issues with inconsistent scheduling, overlapping courses, and stressful timing before exams.
**Remote teaching and flexibility**	Appreciated remote participation options; flexibility during overlapping schedules.	5	Remote teaching was positively received, especially for easing schedule conflicts and improving accessibility.
**Lecture quality**	Lectures were good and informative; teachers were professional; interactive elements like quizzes were appreciated.	5	Overall positive feedback about lecture quality and engagement.
**Course length and structure**	Course was stretched too long; preferred a more compact format and reduce conflict with other courses.	6	A tighter, more concise course structure was suggested for better learning and integration with other studies.
**Student stress and workload**	High stress due to overlapping demanding courses and poor timing of assessments.	4	Students felt overwhelmed by workload and poor scheduling, especially near exams.
**Positive general feedback**	Good course overall; appreciated teacher flexibility; learned a lot despite the challenges.	3	Despite criticism, many students acknowledged the overall value and quality of the course.

In the SBI‐9 questionnaire, that assesses school‐related burnout, the domain score of exhaustion at schoolwork was the highest, with an average median score of 3.8 and a mode of 4 for each of the four questions (mean 3.7) (Table 2). The second highest domain score was found in sense of inadequacy at school with an average median score of 3.5 and a mode of 4 for both questions (mean 3.3). The domain score of cynicism toward the meaning of school was the lowest with an average median score of 2 and a mode of 1 for each of the three questions (mean 2.1).

**TABLE 2:** School Burnout Inventory and distributions of the scores by item (*n* = 49).

Question	Median score	Mode
1. I feel overwhelmed by my schoolwork (EXH1)	4	4
2. I feel a lack of motivation in my schoolwork and often think of giving up (CYN1)	2	1
3. I often have feelings of inadequacy in my schoolwork (INAD1)	4	4
4. I often sleep badly because of matters related to my schoolwork. (EXH2)	4	4
5. I feel that I am losing interest in my schoolwork (CYN2)	2	1
6. I'm continually wondering whether my schoolwork has any meaning (CYN3)	2	1
7. I brood over matters related to my schoolwork a lot during my free time (EXH3)	4	4
8. I used to have higher expectations of my schoolwork than I do now (INAD2)	3	4
9. The pressure of my schoolwork causes me problems in my close relationships with others (EXH4)	3	4
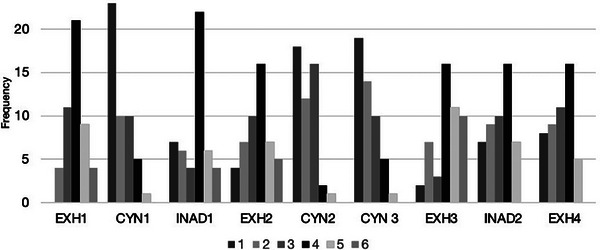		

*Note*: School Burnout Inventory‐9 (SBI‐9) by Salmela‐Aro [15]; EXH = exhaustion at schoolwork; CYN = cynicism toward the meaning of school; INAD = sense of inadequacy at school. The inventory follows a six‐point Likert‐scale from 1 (*completely disagree*) to 6 (*completely agree*). Maximum score of each question is 6 and the total sum score maximum is 54 indicating severe burnout.

## 4 Discussion

Teachers in higher education are required to adapt to evolving technological and institutional demands while maintaining pedagogical methods and support of student engagement and learning [30]. A teacher, as an active agent, strives to influence the learning environment and create optimal learning conditions [31]. All stakeholders in the learning process should be included to improve students’ learning outcomes, and to contribute to the broader advancement of the academic field. At University of Helsinki, the orthodontic course curricula and teaching methods are designed to support the development of undergraduate students into self‐regulated proactive learners. Thus far, only a limited amount of published research data is available on the pedagogical effectiveness of online orthodontic education in enhancing the academic performance of undergraduate students. The current investigation found that, in general, undergraduate dental students prefer a blended teaching approach and the academic performance, as measured by summative examination scores, was equally good in orthodontic theory taught remotely and in‐class.

Our previous pilot study, conducted on the same orthodontics course the previous academic year, found no significant association between examination scores and the mode of teaching or the mandatory nature of the lecture [4]. Our current investigation intentionally employed a different teaching method allocation than the previous study, allowing us to observe the potential impact of course topics on study outcomes [4]. The present findings are, for the most part, in line with our previous ones suggesting reliability and generalizability of the results. However, in the present investigation, each topic was represented by two examination questions and a modest association between the number of students with a correct answer to both questions on each topic and the in‐class teaching method was detected. Therefore, there appears to be potential variation in learning effectiveness of undergraduate orthodontic theoretical instruction depending on the specific modules and topics being taught and the teaching delivery method employed. Further research with a larger sample size is needed to clarify the observed effect of teaching mode on individual student's learning.

Previous investigations of different teaching methods on undergraduate orthodontic courses have shown that both blended and traditional classroom lecture teaching are effective in terms of short‐term knowledge gain [4, 9]. Undergraduate dental students have evaluated that, in learning cephalometrics, blended learning is more likely than either traditional classroom teaching or online learning alone, to be effective [30]. In contrast, orthodontic emergencies taught to undergraduate students via video and flipped classroom method resulted in comparable examination performance and improved levels of satisfaction [32]. Consistent with the literature, this study found that the theory behind conducting a cephalometric analysis was effectively taught remotely, as evidenced by the highest number of correct examination responses to related questions. Overall, based upon our findings, we propose that, with appropriate adaptation of teaching materials, topics that focus on theoretical knowledge and treatment planning could be successfully taught remotely, while specific practical skills and attitudes would be challenging to teach fully online.

The significance of student engagement in educational achievement is widely recognized [33]. Learning analytic interventions, such as the Moodle online track progress function applied in this orthodontics course, may be most effective when targeted to maximize student performance, as suggested by Sonderlund and co‐workers [34]. Findings of previous investigations among university students suggest a positive association between academic performance and online activity in additional resource use [35, 36]. In our study, positive correlation, although statistically insignificant, between examination scores and Moodle activity was also detected implying that actively engaged students score better in examination. This conclusion is further supported by our finding that the examination score of an individual student did not correlate with lecture attendance frequency suggesting that despite limited attendance in the in‐class lectures, students with high level of engagement may achieve strong learning outcomes through self‐directed study and the additional online educational materials.

A significant association has been documented between students' overall mental effort and negative affect that is feelings of emotional distress [37]. Intense mental effort can lead to considerable stress, frustration, and burnout [37]. Burnout is characterized by three key dimensions: increased emotional exhaustion related to work stress, increased depersonalization reflected as negative and cynical attitudes, and reduced feelings of achievement. Subsequently, burnout symptoms are common among medical students [25, 38]. Nearly half of medical students experience symptoms of burnout, with exhaustion being the most prevalent category reported among both medical and dental students [25, 39]. Furthermore, prevalence of depression, anxiety, and stress has been noted to be higher in dental students compared to the general population [40]. Nevertheless, despite high academic burnout frequency, medical and dental students display elevated resilience scores, signifying proficient coping strategies [39]. The students in our cohort were attending three other courses simultaneously to the studied orthodontics course. This increased the risk of cognitive overload and cumulative stress, both of which may contribute to higher levels of burnout. The qualitative analysis of the open‐ended feedback revealed frequent reports on time management challenges.

Salmela‐Aro reported a SBI‐9 score range of 2.4–2.6 in exhaustion category, among Finnish university students, depending on gender and the study year. Cynicism category scores ranged between 2.1 and 2.7 and feeling of inadequacy category scores between 3.1 and 3.7 [22]. Females and students in their final study years displayed higher scores in all categories. Our findings were higher in exhaustion category but similar in cynicism and feelings of inadequacy. We found above scale average level of exhaustion at schoolwork and sense of inadequacy at studies, while the level of cynicism toward the meaning of studies was below the scale average in our group of undergraduate dental students.

Emotional exhaustion, a core component of burnout, is significantly associated with lower grades and decreased study motivation, and the influence is bidirectional [23]. In addition, burnout profiles of individual university student differ according to study success [18]. Students, who score high in exhausted and burned‐out categories of the SBI‐9 have been shown to represent surface learning approach in comparison to students who experience less burnout symptoms [18]. In our study, due to privacy protection of the respondents, no analysis on the individual SBI results could be conducted. Rather, burnout was considered a potential confounding factor that could impact students’ ability to concentrate and thereby affect their academic performance. On a group level, our findings suggest that third year undergraduate dental students find their educational experience and/or life situation to be somewhat stressful.

The main limitation of this small sample study is that the allocation of the lecture delivery modes was not randomized which introduces a possibility of allocation bias. Furthermore, a possibility remains that students adjusted their behavior as a result of being aware of the ongoing study. Another limitation is that burnout symptoms were not followed individually and longitudinally across the course. Therefore, no conclusions on the association between learning, academic achievement, and severity of burnout symptoms could be made. In addition, self‐reported measures can be subject to recall bias [41]. Similar to learning analytics, inventories on burnout symptoms provide opportunities to assist learners, while also posing ethical implications. In this study, the SBI‐9 questionnaire was not repeated, and the results were not used to profile individual students as excessive monitoring and data collection might undermine students' sense of autonomy and agency, making them feel overly scrutinized and potentially negatively affect their learning experience. A potential source of uncertainty related to the questionnaire results is the use of varying response formats. Both Likert scales and categorical options were included. In addition, the wording of the items might be considered somewhat ambiguous, which may introduce interpretive variability among respondents.

Future cross‐sectional studies should examine the association between academic performance, study habits, and the level of burnout in individual level in students willing to partake in such a study. Stress management skills and social support that could moderate the burnout–performance relationship could also be objectives of future studies. By examining burnout and academic performance together, educators can design targeted interventions that aim to reduce burnout while supporting academic success.

## 5 Conclusions

The findings of this study indicate a preference among the majority of undergraduate dental students of a blended teaching approach. Remote teaching of a theoretical orthodontics course yielded equally good overall learning outcomes as in‐class teaching, as assessed by summative examination results. In addition, half of the students reported that blended learning alleviated their study load. The students benefitted on additional online learning material provided, and one third of them considered that the additional material supported their learning process. Our findings imply that the undergraduate dental students of our cohort were experiencing significant stress and self‐doubt related to their academic workload, suggesting a high mental burden. However, their relatively low levels of cynicism toward the meaning or value of their studies indicate that they still found significance in their education, which might act as a protective factor against burnout. The insights gained from this study are instrumental in the development of learner‐centered orthodontic curricula that emphasizes student well‐being in undergraduate dental education. Incorporating flexible learning options, such as blended teaching, can support students at high risk of burnout and promote students’ autonomy and control.

## Conflicts of Interest

The authors declare no conflicts of interest.

## Supporting information




**Supporting File 1**: jdd13995‐sup‐0001‐SuppMat.docx

**Supplementary Table**. List of lecture topics and lecture presentations.

## Data Availability

On reasonable request, the research project's data are made available.
