# Supplementary material for: Student Preference on Teaching Mode and the Impact of Remote Teaching on Academic Performance in Undergraduate Orthodontics Course, a Follow‐Up Study
Source: J Dent Educ. 2025 Jul 16;90(4):524–32. doi: 10.1002/jdd.13995 (PMC13077626; doi:10.1002/jdd.13995)
Supplement: Supplementary file 1 — Supporting File 1: jdd13995‐sup‐0001‐SuppMat.docx [file JDD-90-524-s001.docx]

## Supplementary material

**Feedback questionnaire**

Please choose the alternative that best describes your opinion on the following scale for each question:

Completely disagree, Partly disagree, Neither disagree or agree, Partly agree, Completely agree, I don’t know.

1. Learning goals were clearly defined
2. Teaching modality supported my learning
3. Teaching material supported my learning
4. Course evaluation methods assess well my learning
5. The supplementary interactive material in the Moodle learning platform supported my learning
6. The workload of the course was: Too much, reasonably much, suitable, reasonably light, too light
7. My learning is best supported by: Classroom teaching, Remote teaching, Blended teaching
8. Wy studying workload is reduced by: Classroom teaching, Remote teaching, Blended teaching, I don’t know

Please choose the alternative that best describes your situation (estimation from previous month) on the following scale for each question:

Completely disagree (1), Partly disagree (2), Disagree (3), Partly agree (4), Agree (5), Completely agree (6).

1. I feel overwhelmed by my schoolwork (EXH1)
2. I feel a lack of motivation in my schoolwork and often think of giving up (CYN1)
3. I often have feelings of inadequacy in my schoolwork (INAD1)
4. I often sleep badly because of matters related to my schoolwork. (EXH2)
5. I feel that I am losing interest in my schoolwork (CYN2)
6. I’m continually wondering whether my schoolwork has any meaning (CYN3)
7. I brood over matters related to my schoolwork a lot during my free time (EXH3)
8. I used to have higher expectations of my schoolwork than I do now (INAD2)
9. The pressure of my schoolwork causes me problems in my close relationships with others (EXH4) Note.

EXH = exhaustion at schoolwork; CYN = cynicism toward the meaning of school; INAD = sense of inadequacy at schoolwork
