# Supplementary material for: Student Preference on Teaching Mode and the Impact of Remote Teaching on Academic Performance in Undergraduate Orthodontics Course, a Follow‐Up Study
Source: J Dent Educ. 2025 Jul 16;90(4):524–32. doi: 10.1002/jdd.13995 (PMC13077626; doi:10.1002/jdd.13995)
Supplement: Supplementary file 2 — Supplementary Table. List of lecture topics and lecture presentations. [file JDD-90-524-s002.docx]

Supplementary Table. List of lecture topics and lecture presentations.

| **Teaching duration** | **Mandatory** | **In-class** | **Remote** | **Additional learning material provided** | **Lecture Title** |
| --- | --- | --- | --- | --- | --- |
| 90 | x | x |  | x | Development of occlusion in primary and mixed dentition |
| 90 | x | x |  | x | Tooth development stages, dental age |
| 90 | x |  | x | x | Dental and skeletal malocclusions |
| 90 | x |  | x | x | Cephalometrics |
| 20 | x |  | x | x | Orthodontic impressions (video recording of the lecture) |
| 45 |  |  | x | x | Lingual and palatal arches, and facebows |
| 45 |  |  | x | x | Structure, use and biomechanics of fixed orthodontic appliances |
| 90 |  | x |  | x | Treatment of crossbites |
| 75 |  |  | x |  | Treatment of dental crowding |
| 75 |  | x |  | x | Tissue changes in orthodontic treatment |
| 90 |  | x |  |  | Distal occlusion and its treatment |
| 90 |  |  | x |  | Deep bite and its treatment |
| 90 |  |  | x |  | Open bite and its treatment |
| 90 | x |  | x |  | Missing and impacted teeth: treatment principles and methods |
| 90 |  | x |  | x | Problems related to orthodontic treatment and their prevention |
| 90 |  | x |  |  | Retention in orthodontics |
| 90 | x | x |  |  | Orthodontic treatment assesment: What should be treated in public dental care? |
| 90 | x | x |  | x | Screening patients selected for orthodontics |
| 90 | x | x |  |  | Initial examination in orthodontic treatment |
